# Acquisition of a Transparent Gender System: A Comparison of Italian and Croatian

**DOI:** 10.3389/fpsyg.2020.571674

**Published:** 2020-11-10

**Authors:** Marta Velnić

**Affiliations:** Department of Language and Literature, NTNU-Norwegian University of Science and Technology, Trondheim, Norway

**Keywords:** gender acquisition, gender agreement, gender transparency, Italian, Croatian

## Abstract

Gender transparency is considered a key facilitator for early acquisition of this category. Here, we compare the acquisition of the gender system of two transparent systems, Italian and Croatian. The study focuses on the different degrees of transparency between the two languages by taking into account their extended nominal paradigms. We have conducted an adjective elicitation task on a total of 60 monolingual Italian and Croatian children divided in two age groups (Italian = 3;0 and 3;10, Croatian = 2;10 and 4;2). The results reveal that the Italian gender system is mastered already by the youngest child (age = 2;6) and that the two gender values are acquired simultaneously. However, the Croatian children show a significant difference in the error ratio between the two age groups, which indicates that the gender system is not yet acquired in the younger group (average age = 3;0). Additionally, the results suggest that feminine is the first gender to be mastered in Croatian due to the regularity of its paradigm, and that neuter is the most problematic gender for the children, likely due to its lower frequency and syncretism with masculine throughout the case paradigm. This paper adds to the body of research indicating that transparency of the gender system is not merely a binary feature, it underlines the relevance of placing the languages on a continuum with respect to transparency in order to make predictions related to the acquisition of the gender system.

## Introduction

The aim set forth in this article is to compare how the level of transparency of a gender system influences its acquisition. We will do so by comparing how Italian and Croatian monolingual children acquire their gender system and the individual gender values therein.

Grammatical gender is an inherent property of the noun reflected in agreement with other elements (i.e., determiners and adjectives; Corbett, 1991). A transparent gender system entails that the gender of the noun is evident from its lemma, as nouns have a gender assigned as it is a part of their lexical entry (Kupisch et al., 2002, p. 109). Both Italian and Croatian have transparent gender systems (Kovačević et al., 2009; Gudmundson, 2010). Nevertheless, languages diverge from transparency to some extent (Audring, 2014, p. 6) which means that transparency is not a categorical property, i.e., a binary distinction between a transparent and opaque system. Rather, it should be placed on a continuum.

The different degree of transparency between the two languages will be defined by taking into consideration (i) the complexity of the system, expressed through syncretism, (ii) the number of gender values, and (iii) regularity of gender agreement.

In relation to our first point, the declension classes of the nouns in each language (i.e., number for Italian, number and case for Croatian) have been taken into consideration in order to account for syncretism. Syncretism is defined as the relation between two or more words in a paradigm that have different morphosyntactic features but are identical in form (Matthews, 2007) and this adds to the complexity of the system as having identical forms reduces the likelihood that the markers will be unambiguous (Audring, 2014, p. 12). The number of gender values is also a factor related to the complexity of a gender system (Audring, 2014, p. 14), and may also contribute to the transparency of the system. Croatian has three gender values (masculine-M, feminine-F, and neuter-N) while Italian has two (M and F). In Croatian the three gender values are not equally well represented in child directed speech (CDS) with the neuter amounting to only 6% of nouns (Kovačević et al., 2009, p. 161), whereas no such discrepancy is noted for Italian. Italian has obligatory gender-marked articles that occur with the noun and these are defined as the first and most frequent syntactic cue for gender, thus enhancing the transparency of the language (Chini, 1995). Croatian does not have articles and thus nouns are often bare. We considered these factors for the comparison of the relative transparency of the languages.

We tested the acquisition of gender through agreement by testing children on an adjective elicitation task. Adjectives were chosen as these are optional and gender-marked in both languages and were therefore considered a comparable testing ground. The results have shown considerable differences between the two language groups, and we discuss this based on the different degrees of transparency.

The paper is structured as follows: in the next section we provide a general overview of the acquisition of gender systems, in section “The gender systems of Croatian and Italian” we move on to describing the genders systems of the two languages, with a subsection on previous research on gender acquisition for each language. In section “The Current Study,” we describe the aims of the current study and lay out the research questions, followed by the methodology section. Section “Results” focuses on the results, followed by a discussion (section “Discussion”) and conclusions (section “Conclusion”).

## The Acquisition of Gender: A General Overview

From the perspective of language acquisition, there are two types of cues that contribute the acquisition of gender: formal and semantic cues. The former are morpho-phonological cues that appear on the noun, the latter correlate to the natural gender of the referent. Research on various languages, such as Hebrew (Levy, 1983), French (Karmiloff-Smith, 1981), Russian (Rodina, 2008), and Spanish (Pérez-Pereira, 1991) has shown that children rely more on formal than semantic cues. Hence, these will not be discussed further. Previous studies have correlated the time course in the acquisition of the gender system with its transparency: if the gender assignment system is transparent it will be acquired more easily; as this property facilitates early use of correct agreement (Kupisch et al., 2002, p. 141; Rodina and Westergaard, 2015, p. 197). On the other end of the spectrum, in languages with opaque gender systems, this property is acquired late, such as in Norwegian (Rodina and Westergaard, 2015, p. 200).

Formal cues include both phonological and morphological assignment systems. The latter needs to refer to more than one form of the noun, i.e., a declension class, whereas gender assignment in the former is evident from a single nominal form (Corbett, 1991). As one of the reviewers pointed out, the two formal systems may also overlap, and this adds to the transparency of the system^1^. Thus, if the formal cues are clear, i.e., transparent, children should acquire them more easily. Sá-Leite et al. (2020) emphasize the role of transparency in gender acquisition stating that it diminishes the role of agreeing elements in gender awareness and its assignment (p. 693). In this study we consider a gender assignment system to be transparent when the formal assignment of gender allows for an accurate inference of the gender of the noun without having to rely on agreement on other arguments; as a high complexity of the nature and number of assignment rules may lead to a greater difficulty for the acquiring child (Audring, 2014, p. 16).

Spanish is a very clear example of a transparent system: the final vowel of the noun predominantly signals its gender: *-o* for M and *-a* for F, i.e., *armario*-M ‘closet,’ *mesa*-F ‘table’ (Pérez-Pereira, 1991); and this is true also for some referents with a semantic gender, i.e., *chico*-M “boy” and *chica*-F “girl.” Conversely, Germanic languages have mostly opaque systems as the noun itself offers no, or very little, indication of the gender, take Norwegian as an example: *stol*-M ‘chair, *seng*-F ‘bed,’ and *skap*-N ‘closet’ (Rodina and Westergaard, 2015). The number of gender values is also a factor which contributes to transparency: a two-way gender system should be easier to acquire than a three -way gender systems. Nevertheless, Rodina et al. (2020) specify how formal transparency seems to be more important as transparent three-way gender systems (i.e., Greek) are known to be acquired before opaque 2-way gender systems such (i.e., Dutch).

Gender systems have an unmarked gender value; this is usually M. This is the case for Spanish and Portuguese, as in case of erroneous assignment children tend to attribute F nouns to M in Spanish (Pérez-Pereira, 1991); whereas in Portuguese, 2 and 4 year-olds were found to alter novel F nouns to match the gender of the determiner more often than they did with M nouns (Corrêa et al., 2011).

Differently from nouns, other arguments receive gender through agreement (Kupisch et al., 2013, p. 153). A rich agreement system facilitates acquisition as languages in which a lot of elements agree with the noun (e.g., Spanish, French) is mastered earlier than a system with few agreement markers, such as Dutch (Audring, 2014, p. 16). In the current study we use agreement (of the adjective) to establish gender acquisition, as has been done in previous studies (Karmiloff-Smith, 1981; Kupisch et al., 2002; Rodina, 2008).

The key premise of this study is that transparency is not a binary feature and comes in degrees. Previous studies have found pivotal differences in the acquisition of two languages with fairly similar gender systems. Smoczyńska (1985) compared Polish and Russian, and found that the Polish gender system is more easily acquired. The reason for this is the different degrees of transparency between the two languages. Russian has two classes of opaque nouns, as defined by Rodina and Westergaard (2017): nouns ending in a palatalized consonant which may belong either to the M or the F gender, and N nouns ending in an unstressed -*o* are indistinguishable from the typical -*a* ending of F nouns making the nouns in question ambiguous between F and N.

An effect of the degrees of transparency was also found between French and Italian as gender in Italian is acquired earlier than in French (Kupisch et al., 2002, p. 116) because the formal regularities are more reliable in Italian. The French system is not considered opaque, as in both languages gender is unambiguously marked only on determiners, but Italian has a more reliable morphology (Kupisch et al., 2002, p. 108). Kupisch et al. (2018) have thus put forth a continuum of gender transparency that places these languages in the context of others:

Spanish > Venetian > Italian > Russian > French > German > Norwegian/Swedish/Dutch

Additionally, a thorough analysis of previous research on gender acquisition and processing by Sá-Leite et al. (2020) has found that Romance languages generally fall into the transparent side whereas Germanic languages tend to be placed on the opaque side of the continuum (p.693). They also add that the picture is less clear for Slavic languages which is why research on gender is Slavic languages is relevant. Similarly, Rodina et al. (2020) divide languages into three categories of transparency: Type-I languages provide unambiguous cues for a particular gender and the agreeing elements are often phonologically similar or identical to the assignment markers and nouns are rarely bare; Type-II languages are defined as semi-transparent as they contain both highly transparent and ambiguous cues; in Type-III there are hardly any formal or semantic cues, with articles providing some structural cues; children acquiring gender in these languages struggle throughout their preschool years (Rodina et al., 2020, p. 3). They classify Spanish and Italian as Type I, Russian, Latvian, Hebrew, and German as Type II, and Dutch and Norwegian as Type III. Thus, there is a general consensus that Romance languages are transparent, while Germanic ones are opaque, with the rest of the languages that were studied falling somewhere in between. Nevertheless, this is not a clear-cut division as we can see French (Romance) being categorized as less transparent than Russian (Kupisch et al., 2018), and German (Germanic) classified as semi-transparent (Rodina et al., 2020). In the following section the gender systems of Italian and Croatian will be thoroughly explained, and we will propose where Croatian might be placed on this scale.

## The Gender Systems of Croatian and Italian

In the following sections we will describe the gender system of Italian and Croatian in order to outline the level of transparency of each language.

### The Italian Gender System

The Italian gender system has two values, M and F, and gender is expressed through morphophonological properties of the noun ending (Chini, 1995). The distribution of the two gender values in the language is 60% of M nouns and 40% of F nouns (Costa et al., 2003, p. 186). Gender assignment is mainly morphological as the noun endings are morphemes that change based on number, i.e., *libro/libri* (book/s), and the gender is assigned in function of the nouns declension class, which depends on the singular/plural paradigm^2^. Nevertheless, as is displayed in Table 1, phonological transparency is also high in Italian since the *-o* and *-a* for singular are the most frequent noun endings; these, according to De Martino et al. (2017), signal respectively M 99.97% of the time and F 99.9% of the time.

**TABLE 1:** The Italian declension classes.

**Noun class**	**Gender**	**Ending sg/pl**	**Example**	**Translation**	**Frequency in LIP (%)**
A	M	-o/-i	Libro/i	Book/s	39,80
B	F	-a/-e	Padella/e	Pot/s	31,40
C1	F	-e/-i	Volpe/i	Fox/s	21,49
C2	M	-e/-i	Cane/i	Dog/s	
C3	M&F	-e/-i	Insegnate/i	Teacher/s	
Other	M&F	-á/-á; -a/-i, etc.^a^	Abilità, Problema/i	Ability	7,30

*^*a*^The omitted endings all had a representation in the corpus lower than 1%*

According to Noccetti (2002), M is the unmarked gender, and deriving F nouns is possible via suffixations for some animate nouns (*lupo*-M “wolf”/*lupa*-F “she wolf,” but not *uccello*-M “bird”/^∗^*uccella*-F). Thus, similarly to Portuguese (Corrêa et al., 2011), the *-a* in F nouns such as *sedia* is a thematic vowel, but it is a gender marked form of an inflectional morpheme in cases like *lupa*.

Italian nouns can be divided in declension classes based on the noun endings in the singular and plural. The declension classes that make up the majority are displayed in Table 1, along with the frequencies from the LIP corpus^3^ (Voghera et al., 2014). The table is a summary from Gudmundson (2010), we use the same terminology as the author.

Classes A and B are the transparent classes as the -*o/-i* and *-a/-e* endings unequivocally signal M and F respectively. However, there are numerous declension classes (Gudmundson, 2010 lists 16) each with a unique combination of sg/pl endings, so how can this system be considered transparent? Gudmundson’s corpus analysis of the LIP corpus (Voghera et al., 2014) revealed that the two groups of transparent nouns constitute 71.2% of tokens used. The third most frequent noun class amounting to 21% of the corpus consisted of the ambiguous *–e/-i* class. Nevertheless, this class can be broken down into groups based on derivational morphemes, some of which have been found to unambiguously signal the gender of a noun: -*ione* (F) (*stagione-* “season”), *-tore* (M) and *-trice* (F) respectively (*attore/attrice*- “actor/actress”), *-iere* (M) (*giocolliere-* “juggler”), *-ame* (M) (*fogliame*- “foliage”), and *-udine* (F) (*solitudine*- “loneliness”); according to Gudmundson (2010) these account for 52% of nouns in class C. Thus, even if the majority of noun classes in Italian can be classified as opaque, when the size of the groups and the nouns that appear there in is considered together with frequency, it is evident that the majority of the nouns (∼82%) are transparent (Gudmundson, 2010, p. 14).

The gender of the opaque nouns becomes explicit through agreement, thus the rich inflectional system of the nominal domain in Italian should be a valuable resource for the acquiring child. Elements that have gender agreement are articles, determiners, adjectives, quantifiers, possessives, *wh*-words, relative clauses and the past participle (Chini, 1995; Gudmundson, 2010). Here, we will focus on the description of the article and adjectival systems. The article is the first and most frequent agreement cue for gender, moreover, it is the only cue when the noun does not have transparent assignment (Chini, 1995, p. 93), while the latter is relevant because we used adjectives as test items in our task. Chini (1995) defines the paradigm of M article as more complex than that of the F article, because (i) it contains more allomorphs (*il* vs. *lo*) the choice of which depends on the first phoneme of the noun, and (ii) because the unmarked form for *il* does not end in *-o* as the typical M nouns do. Adjectives also agree in gender with the noun and their ending morphemes are equivalent to A and B declension classes in Table 1, taking *-o/-i* for M and *-a/-e* for F (*il libro bello*- the beautiful book/*la sedia bella^4^ - the beautiful chair*). There are exceptions and some adjectives do not vary for gender but keep the number distinction, these have *-e/-i* endings as the C-class nouns (*il libro verde*-the green book/ *la sedia verde*- the green chair), while some adjectives do not vary for number and gender at all (i.e., *rosa*-pink).

Summarizing, the majority of Italian nouns have a transparent gender assignment, but because of obligatory article inflected for gender even these nouns are disambiguated.

#### The Acquisition of the Italian Gender System

It has been reported that the Italian gender system is acquired in a short time and with very few errors in the article system (Kupisch et al., 2002, p. 117). When it comes to the distribution of declension classes in child speech and CDS, De Marco (2005) found that the two transparent classes are the most represented ones, with nouns of what we referred to as the C-class being less frequent. No reports on the other, much less frequent declension classes, are made in that study. Thus, De Marco (2005) found that A and B-class nouns (singular) amount to 63% in CDS (average across the corpus), and to 66% in child speech; whereas the C-class nouns are produced at 11 and 15%, respectively, by each group. Noccetti (2002, p. 62) also reports that the A and B declension classes (to which she refers to as *productive microclasses*) are the most frequent ones both in CDS, in the child’s utterances, and also in adult to adult speech.

When it comes to the opaque nouns, various studies (Caselli et al., 1993; Kupisch et al., 2002; Noccetti, 2002; Belletti and Guasti, 2015) report that also these are acquired easily. A corpus investigation conducted by Kupisch et al. (2002, p. 115) revealed that 90% of all noun tokens belong to the two noun classes defined as transparent, and additionally, in CDS, diminutive suffixes are used rather often which makes the gender of the noun available also on C class nouns (i.e., *il cane/il canino*- “the dog/doggie,” *la volpe/la volpicina*- “the fox/foxy”). The Italian monolingual corpus investigated by Kupisch et al. (2002, p. 117) contained very few gender errors and they conclude that gender in Italian is mastered early without significant deviations from the target gender. Noccetti (2002) provides a more detailed analysis of a corpus of a single child and discusses a more sequential acquisition of Italian gender. In the *premorphology phase* (2;00–2;3) of gender acquisition the child makes some assignment errors (i.e., gender mistakes in the plural) and the article is omitted. In the *protomorphology phase* (2;3–2;8) mistakes in agreement of C-class nouns are present as they are generalized to M; however, the B-class nouns (F gender) are productive, for example the child pluralized a proper name. In the *modularized morphology phase* (2;8–3;4), the child uses the paradigms and agreement of all genders correctly. Thus, Italian children go through stages in the acquisition of Italian gender, but we can see from Noccetti’s (2002) data that these phases are quite brief which is probably why there were not observed by other, perhaps less detailed, studies.

According to Kupisch et al. (2002, p. 116) articles have a relevant contribution for the early acquisition of gender in Italian, because transparency reinforces the early acquisition of articles, and once they are acquired it is an additional facilitation for the acquisition of gender. In a study aiming precisely on adjectives, Bottari et al. (1993) analyzed the production of monosyllabic place holders that emerge before lexical items and that the correct morpheme *la* form the F article is realized before its M counterpart. They explain this in relation to *la* having vowel features analogous the noun, which the M article does not have. This is strengthened by the fact that the plural article *le* is also learned before *il*, in spite of the lower frequency of the plural in the input. Similarly, Pizzuto and Caselli (1992) found based on corpus data of three children that the F article *la* is the first one to be attested (age = 1;6). The errors of commission observed in the study are related to the phonological choice of M article of choosing *lo* over *il* (Pizzuto and Caselli, 1992, p. 540). Pizzuto and Caselli’s (1992) analysis shows that even if the articles are attested early, only *la* can be considered acquired by all three children in the corpus. Moreover, what they conclude is that none of the major inflectional paradigms that they investigated (articles, pronouns, clitics, and verbs) is fully mastered by the age of 3;0. Thus, article as a gender cue certainly contributes to acquiring the gender system, it does not entail that the system is immediately mastered. Therefore, investigating whether gender is productive on agreeing elements other than articles can give us insight into when the system is acquired. For example, Caselli et al. (1993) conducted an elicitation task on three age groups and found that *la* has a better accuracy than *il* in the youngest group, but agreement on adjectives is at ceiling in all three age groups (Caselli et al., 1993, p. 384).

We can see from the reported studies that there is an early mastery of the Italian gender system, even for the classes that are deemed less transparent, because of a frequent gender cue in the form of an article. It would seem that for articles the F article *la* is acquired with more ease, but this does not entail that the F system as a whole is mastered before M. The latter is considered the unmarked gender and studies on other Romance languages have found that it is the unmarked gender that is acquired more easily (Pérez-Pereira, 1991; Corrêa et al., 2011).

### The Gender System of Croatian

Croatian has three gender values: M, F, and N. An investigation of the 4000 most spoken (nouns) lemmas in adult spoken language revealed that M nouns amount to 43,3%, F to 42,9%, and N takes up the remaining 13,7% (Vuletić, 1991) in Kovačević et al. (2009, p. 157). Croatian can be considered a system with morphological gender assignment because the assignment rules require access to more than one form, in our case the declensional class, similarly to Russian as defined by Corbett (1991, p.36). The morphological properties of the noun act as a reliable cue for gender. Considering the NOM.SG, M nouns end in a *-ø* morpheme and thus in a consonant (*konj* “horse,” *prozor* “window”); F nouns end in *–a*, (*kuća* “house”); and N nouns end in *–o* or *–e*, (*stablo* “tree,” *more* “sea”). However, some exceptions apply to M and F as there are M nouns that end in an *-o* (*pepeo* “ash”) but also in an *-a* (*gazda* “boss/owner”); and some F nouns end in a consonant (*kost* “bone) (Barić et al., 2005).

Unmarked options are usually defined by the underspecification of features (Halle and Marantz, 1994; Carstairs-McCarthy, 1998). Thus, M is the unmarked gender, as M nouns end in a consonant or a -*ø* morpheme, and other morphemes are added to this root when the noun is declined by case (*konj*-NOM/*konj-a*-ACC), whereas for F nouns, the *-a* ending can be considered a morpheme as it is absent in the subsequent declinations of the paradigm as it is replaced by another morpheme (*kuć-a*-NOM/*kuć-u*-ACC/^∗^*kuća-u*).

When it comes to the relation of other morphemes and their gender transparency, the derivational morpheme -*ica* is very productive in forming F animate nouns from M roots: *učitelj*-M (teacher), *učiteljica*-F (female teacher). The morphemic endings are constant across derivational morphemes, such as diminutives which end in -*ić* (*konjić* -M), -*ica* (*kućica*-F), and -*ce* (*stabalce*-N). The semantic core of nouns is also frequently compatible with the described morphology: *muškarac*-M (man), *žena*-F (woman/wife), *dijete*-N (child) which should contribute to the overall transparency of the system.

Croatian has three declension classes, but these do not match the three gender values. The criteria for grouping the nouns in declension classes are based on the ending that the noun takes in the genitive singular: class *-a*, class *-e*, and class *-i*. The first class includes M and N nouns, the second class includes mostly F nouns with some M exceptions such as *gazda* “boss/owner,” while the *-i* class contains only F nouns^5^, the ones ending in a consonant (Barić et al., 2005, p. 103). Thus, F nouns span over two classes, whereas M and N share a declension class.

In Table 2 we present the full paradigm of Croatian declension. This includes the three declension classes divided into their gender values^6^.

**TABLE 2:** The Croatian declension classes.

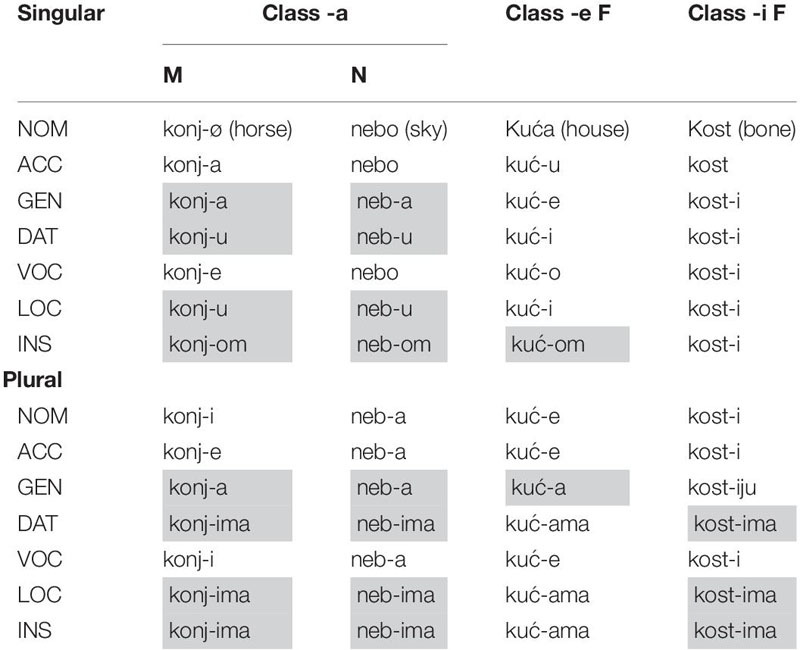

The overwhelming syncretism between M and N (shaded in gray) is obvious from Table 2. This is not surprising considering that these two genders are part of the same declension class. In this kind of system, the declension class is not really informative for gender and this affects the overall transparency of the system as the gender of a noun is opaque when expressed in these cases. The syncretism of the *-ima* (pl) ending also extends to the F *-i* stem declension class in DAT, LOC, and INS case. The F nouns from class *-e*, included in this study, are only syncretic (same form and function of the suffix) with the other two gender values in INS.SG and GEN.PL The table also shows how the F nouns in class *-e* have also some other shared suffixes as the suffix *-a* is shared by NOM.F and ACC.M, the suffix *-u* is shared between DAT.M and ACC.F, *-e* is shared between VOC.M and GEN.F. But since words are rarely produced in isolation, it will be obvious from the context, for example, whether a word is in DAT or in ACC case.

When it comes to agreement, demonstratives, possessives, adjectives and the periphrastic past tense show gender agreement. In (1) we can see the demonstrative agreeing with the M, the possessive with the F, and the adjective with the N noun. However, since these are not obligatory elements, the Croatian noun is often bare, with only the morphology of the noun itself serving as a gender cue; like in (2).

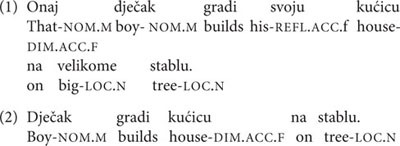

Regarding the agreement paradigm, we will outline only on the adjectival one because adjectives were elicited in our task. Adjectives agree with gender and are not dependent on the declension class of the noun, the morphemic endings are as the NOM.SG (*Lijep(i)-M konj*^7^ - Beautiful horse*/lijepa-F kuća* – beautiful house/*lijepo-N stablo* – beautiful tree). They can appear both in attributive and predicative position, both of which are gender-marked.

Overall, the gender system of Croatian is transparent as the noun endings in the nominative singular quite unambiguously indicate the gender value of the noun; also, the agreeing elements display a transparent gender marker. However, when the whole nominal paradigm is taken into consideration, the initially straightforward transparency becomes more limited: the M and N genders are revealed as much closer than initially shown by their declension in the nominative case, as syncretism of the declension is present in four out of the seven cases. The most transparent gender is F, as the syncretism it has with the other genders is within case only in INS in the nominal paradigm and never within case in the adjectival paradigm. However, the 3rd declension class of F opaque nouns contributed to the reduced transparency of the system overall, and even if these nouns are excluded from the current study this has to be taken into account for pacing Croatian on the scale. We know from Russian that nouns ending in a palatal consonant are ambiguous between M and F and are often overgeneralized to M in monolingual speakers during the preschool years (Rodina et al., 2020).

#### The Acquisition of the Croatian Gender System

The acquisition of Croatian is overall understudied, and thus reference to numerous studies is not possible. Croatian is a language with rich derivational morphology, and this morphological richness could act as a booster for the acquisition of the inflectional paradigms (Kovačević et al., 2009, p. 153).

Kovačević et al. (2009) analyzed corpus data from one of the children present in the Kovačević (2004) corpus from ages 1;3–2;8. The study focuses on the child’s incremental acquisition of the case system and only marginally looks at gender, focusing mainly on the use of nouns in relation to the three genders rather than the gender markings on agreeing elements. They noticed that the distribution of the three genders reflects the distribution of the nouns in CDS of the same corpus: 35% M, 59% F, and 6% N. Consequently, the child produced mostly F nouns (58%) followed by M (36%) and N (6%). Other than that, there are no notes on the gender agreement of these nouns, nor are there any indications of how accurate agreement is.

With regard to the acquisition of the case paradigm, the authors state that the first opposition of inflected forms are found in regular F nouns between the NOM and ACC case (Kovačević et al., 2009, p. 172). They find that the child grasps the complex case system easily and is using all seven cases, with different frequencies, at age 1;10. This implies that all the children that participated in our task should be able to use the full declensional paradigm. The corpus results from Kovačević et al. (2009, p. 165) report the nominative and accusative reaching together 78% of the child’s production at age 2;5. This prevalence of the two aforementioned cases is also evident in the input. Recalling Table 2, NOM and ACC are not syncretic at any level, and considering the very low frequency of N nouns (6%), the syncretism between M and N might not be evident from an early age. Since the corpus contains data only until 2;8, evidence of a more distributed use of the case paradigm or a more frequent use of N nouns is not provided. We might assume that the neutralization of M and N becomes more evident with increased exposure and usage of the full case paradigm, and we can also speculate that this might be reflected in the acquisition of gender, namely that it will take children longer to master M and N because it will take them longer to become aware that these nouns belong to different genders. There are no studies that investigate the role of a rich case system in gender acquisition, thus we cannot infer whether a system with many cases will have a positive or a negative effect on early mastery of a gender system. If the entire case system affects the acquisition of gender, a case system with numerous cases might hinder gender acquisition; but if the nominative has the main role for gender acquisition, then the timing of gender acquisition will be affected mainly by the transparency degree of this case.

### Main Differences Between Italian and Croatian

Here we will concisely summarize the crucial differences between Italian and Croatian which are relevant for the predictions that we make (section “The Current Study”). This is visible in Table 3 where the facilitative factors are shaded in gray.

**TABLE 3:** Crucial differences between Italian and Croatian.

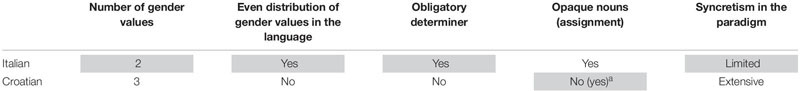

*^*a*^The *-i* class F nouns are opaque, but not part of the current study.*

Italian is the more transparent gender system: it has fewer gender values and these are more evenly distributed, it has an obligatory gender article, and the syncretism of the article is limited^8^. Both languages have a set of nouns that are considered opaque: for Croatian this is the *-i* declension class, but it is excluded from the task in this study giving Croatian a less opaque gender assignment system for the purposes of this particular study, while Italian has nouns ambiguous between M and F (C-class) (Gudmundson, 2010, p. 14). The gender these nouns is, however, clearly disambiguated with the obligatory gender-marked article. The Italian nominal paradigm is more limited than the Croatian one because it does not include case. Thus, when comparing Croatian nouns to Italian ones only in the nominative, the two systems seem transparent to the same degree. However, when all cases in Croatian are taken into account, it becomes clear that M and N are part of the same declension class and thus the paradigmatic differences are often neutralized, leaving F the least ambiguous gender value. Additionally, the Italian M and F values are quite evenly distributed in speech (Costa et al., 2003, p. 186), as they are in Croatian, however, the N here is significantly less frequent than the remaining two gender values (Kovačević et al., 2009, p. 161) which could affect the time that it takes for children to master it.

Going back to the continuum of gender transparency presented in section “The Acquisition of Gender: A General Overview,” we may now place Croatian on this continuum. From what has been outlined in the current section, Croatian is less transparent than Italian and should thus be placed to the right of it. On the scale presented by Kupisch et al. (2018), Russian is placed next to Italian. We deem Croatian more transparent than Russian because Croatian does not have ambiguities in the assignment system of the gender as the ones described by Rodina and Westergaard (2017). The updated scale follows:

Spanish > Venetian > Italian > **Croatian** > Russian > French > German > Norwegian/Swedish/Dutch

Interestingly enough, Italian and Croatian are next to each other on this scale, and thus we will be observing differences in the acquisition of two gender systems with similar degrees of transparency.

## The Current Study

The importance of this study lies in the fact that we are comparing two languages that have transparent gender systems, which has been repeatedly found to be the key to an early acquisition of gender (Levy, 1983). Based on this premise, both Italian and Croatian children are expected to have an early grasp of their respective gender systems.

However, the two target languages differ in other relevant features which may affect the timing of acquisition of the system and the individual gender values, even if gender is acquired early in both groups. We thus expect a high rate of correct agreement, but the intra-language differences are expected to affect the timing of and perhaps the order in which a particular gender is mastered. Thus, the relevance of this study lies in the fact that we are analyzing how different factors in transparent languages can affect gender acquisition. Additionally, the acquisition of the gender system in Croatian is relatively new territory, with only one study taking it into consideration: Kovačević et al. (2009) but not from the perspective of agreement. It will thus offer a starting point for further investigation of the acquisition of gender in Croatian, according to which more specific research questions could be tackled through more precisely designed tasks.

We have decided to elicit adjectives because of their optional status in both languages and the fact that in both languages the declension paradigm is regular and thus offers good grounds for comparison. The study aims to answer the following research questions:

(1) Are Croatian children slower than Italian children to acquire the gender system?
(2) Is the most regular gender (F) acquired first in both languages?
(3) How do the acquisition paths differ in the two languages?

Based on the properties of the two gender systems and what has been found so far in previous studies, we predict that Croatian will be acquired more slowly when compared to Italian. This prediction is primarily based to the syncretic distribution of infections of M and N in some parts of the case system (Table 2) accompanied by the low frequency of N nouns in CDS. Whereas in Italian the presence of the obligatory gender-marked article is a relevant cue and children have been found to acquire gender unproblematically.

Regularity and transparency are relevant factors, and it has been found for Italian articles that *la* is mastered first. Nevertheless, this does not entail that F is overall acquired, and with the default status of M, it might have the upper hand in being acquired first as it similarly happens for Spanish (Pérez-Pereira, 1991). For Croatian, M can also be considered unmarked. However, the F is both more regular and does not have syncretism like M and N do, and this might positively impact the acquisition of F in Croatian.

We expect the three Croatian genders to be acquired in different stages, as we expect the low frequency of N to result in a later acquisition of this gender value.

Overall, this study will compare the acquisition of two easily acquirable gender systems, and it will thus reveal the nuances of how the gradience of gender transparency affects accuracy.

## Methodology

The task consisted of adjective elicitation using images of animals and objects denoting referents of different genders. We have chosen to elicit adjectives since they are optional and agree in gender with the noun in both languages.

### Participants

Two samples of children participated in the research, native speakers of Italian (*n* = 30, 11 male) and native speakers of Croatian (*n* = 30, 16 male). Each language group included two age groups of 15 children each. The children that were chosen for this research were required to be native and monolingual speakers. The parents/caregivers were informed about the testing and had to sign a consent form in order for their child to participate.

Ensuring age balance across the language groups was not entirely possible due to the availability of children and parental consent. The language groups were still close in age and we were able to make relevant statistical comparisons. The mean age of the younger Croatian group (*n* = 15, 6 male) was 2;10, whereas the older group (*n* = 15, 10 male) had a mean age of 4;2. The mean age of the younger Italian group (*n* = 15, 3 male) was 3;0, and the older group (*n* = 15, 8 male) had a mean of 3;10. This means that the age range of the Croatian children was wider than the one of the Italian participants, which was also convenient as we expected the former to acquire gender at a slower pace than the latter. Thus, with a wider age range, we will be able to observe this potential difference more clearly.

Two participants were excluded from the younger groups due to their general lack of adjective production; both children, one from the Italian and one from the Croatian group, were 2;3. This does not automatically suggest that gender is not or cannot be acquired before age 2;6, but it indicates how children are not producing enough adjectives at this age.

### Materials

Thirty images depicting animals and inanimate objects were used. The images were downloaded from the Internet from open source websites and printed on white tick paper. These images were selected on the basis of the grammatical gender of the depicted noun in Italian and Croatian. The selection was proportional, so that we had the same number of items per gender value in each language. This means that, in Italian, fifteen images represent an M noun and the other fifteen represent an F noun, In Croatian, ten are M, ten are F, and ten are N.

The test items were the same in both languages and hence were selected considering their gender values across the two languages. Thus, *tree* was M in Italian, and N in Croatian, *snail* was F in Italian and M in Croatian, and so on. As we went along with the choice of the stimuli, these had to fill specific gender slots in both languages depending on how many were left for each gender. Unfortunately, due to matching the referents to the genders cross-linguistically, the Italian C-class nouns (*n* = 6) are contained only within M. However, as the previous literature has stated, the article is a very reliable marker of gender (Chini, 1995) and Italian children have been found to master these types of nouns easily (Belletti and Guasti, 2015). Due to a considerable amount of N referents denoting abstract concepts, nouns such as *sky* had to be included. We did not explicitly check for frequency of these nouns in the two languages, but during the piloting of the task all the participants were able to name all the images included. The only somewhat problematic item was *wing*, but it could not be replaced due to a low availability of nouns that were N in Croatian and F in Italian. A list of all the items divided by language and gender is displayed in the Appendix.

### Procedure

All the participants were interviewed individually. Interviews were held in an isolated area of the kindergarten in order to avoid acoustic interferences. Interviews lasted from 5 to 15 min. The interviewer explained to each participant that they would have to describe the referents shown on the images by using words like *beautiful, good, small.* The interviewer made sure to use plural agreement, since the elicitation was for singular forms.

When it was clear to the participant that the description had to include an expression of a quality of the shown object, the testing began. The interviewer took a test item from a bag in a random order and then named it, avoiding any agreement. Then the child was asked to describe it. The production itself was not guided in any way and children described the images with an adjective of their choice; which for Italian meant, as we will see in the results section, that adjectives without gender-marking were occasionally used. The interviews were recorded with a digital recorder Panasonic RR-US430 either held in the interviewer’s hand or laid on the desk. The uttered adjectives were manually transcribed on a paper during the interview, and this was then cross-referenced with the recording.

When the child produced incorrect agreement, the question was repeated in order to establish if that was just a simple distraction or if it was a non-target-like gender production. If the child made a correct agreement the second time, the response was considered correct, otherwise it was scored as incorrect.

## Results

Both language groups had a high accuracy rate and we can safely assume that they are aware of the nominal category of gender and use it accordingly. An answer was counted as correct if the adjective gender matched the gender of the noun, it was incorrect if it did not. The non-applicable (NA) category includes the following cases: no response (*n* = 155), no adjective produced (*n* = 54), and use a non-gendered adjective (only for Italian children) (*n* = 35).

In the following sections we will address the research questions laid out in section 4 more specifically. However, for the rest of the analyses we will focus on the binary distinction consisting of correct/incorrect gender agreement, raw results displayed in Table 4.

**TABLE 4:** Distribution of correct/incorrect answers divided per groups.

**Group**	**Target gender**	**Correct**	**Incorrect**
It.young	M	165	/
	F	166	4
It.old	M	205	/
	F	201	/
Cro.young	M	123	8
	F	117	8
	N	110	17
Cro.old	M	137	6
	F	145	1
	N	135	9

### Timing of Gender Mastery

We have conducted a linear regression using Jamovi (Love et al., 2018) on the data excluding NA responses. Here, group (young vs. old), language (Croatian vs. Italian), and target gender (M, F, and N^9^) were set as factors. The outcome of the analysis is presented in Table 5.

**TABLE 5:** Linear regression of all groups.

	**Estimate**	***SE***	***t***	***p***
Intercept	0.95704	0.00504	190.006	<0.001
Old-young	0.03154	0.00904	3.489	<0.001
Cro-It	−0.03623	0.01004	−3.610	<0.001
F-M	0.00138	0.00992	0.139	0.890
N-M	−0.05309	0.01411	−3.763	<0.001

The intercept was set to the values of *young*, *Italian*, and *M*. The significance at the group and at the language level shows that the four groups we tested show significant differences in their responses, however, the nature of these differences is not yet evident from the current analysis. The fact that there is no difference in the responses to the F and M gender means that the children master it to the same extent in both languages; the difference between the N and M refers only to the Croatian group and the results suggest that the children are significantly less accurate with one of the genders. In light of the raw data, it is evident that N agreement is less accurate, but more precise statistical analysis will reveal whether this difference is significant. We also ran a linear regression on the animacy of the test items in order to check whether this is an underlying effect of our results: animacy does not seem to play a role as the correctness of the responses was not significantly different in animate and inanimate test items (*p*-value = 0.146).

The next step in our analysis is to look at the language groups by conducting a linear regression on the Italian and Croatian groups separately, both analyses are displayed in Table 6. This separation will shed light on the source of the significant difference in Table 5.

**TABLE 6:** Linear regressions on Italian and Croatian groups (run separately).

	**Estimate**	***SE***	***t***	***p***
**Italian**				
Intercept	0.9940	0.00269	369.49	<0.001
Old-young	0.0118	0.00538	2.19	0.29
F-M	−0.106	0.00536	1.98	0.48
**Croatian**				
Intercept	0.9384	0.00826	113.652	<0.001
Old-young	0.0491	0.01652	2.971	0.003
F-M	0.0171	0.02017	0.846	0.398
N-M	0.0624	0.02022	3.084	0.002

The statistical analysis did not reveal any significant differences between the two age groups of Italian children and found that both genders are acquired equally well. This means that the Italian children master gender by the age of 2;6, which is the age of the youngest participant taken into consideration. The task does not offer insight into gender mastery prior to 2;6.

Moving on to the analysis on the Croatian groups, there is a significant age difference which means that the correct answers increase significantly with age (*p* = 0.003). Again, there is no difference between M and F, but there is a significant difference between M and N (*p* = 0.002). This suggests that N is acquired later than the other two genders in Croatian. In order to check for this, we have to make further analyses. We have thus tested another dataset from which we excluded N (Table 7). If the difference in the age groups is no longer significant, it would mean that the low accuracy of N is the sole reason for the observed difference in Table 6.

**TABLE 7:** Linear regression of the Croatian groups with no neuter gender.

	**Estimate**	***SE***	***t***	***p***
Intercept	0.9567	0.00860	111.18	<0.001
Old-young	0.0380	0.01721	2.21	0.028
F-M	0.0172	0.01718	1.00	0.316

The analysis showed that the difference between the younger and older children is still significant, but to a lesser degree, when N is not accounted for. This means that N strongly contributes to the age difference of Croatian children, but it is not the only factor and Croatian children acquire gender at a slower pace than Italian children do (statistical difference between the two age groups not observable for the Italian group).

### The Acquisition of F

**FIGURE 1:**
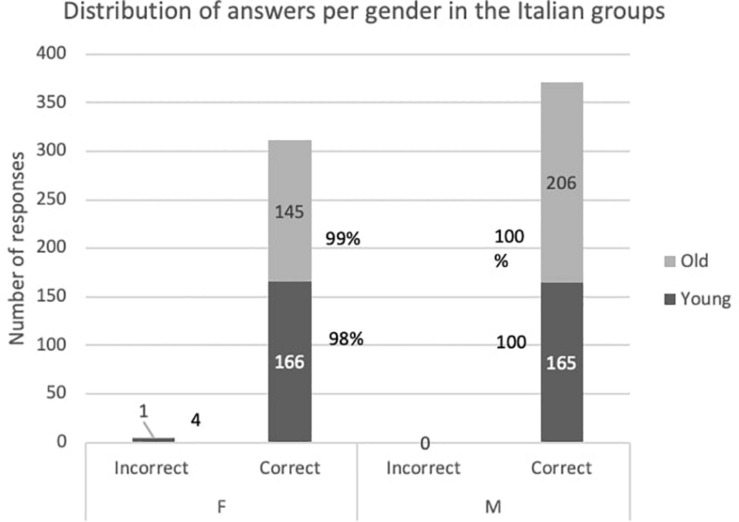
Distribution of answers per gender in the Italian groups.

**FIGURE 2:**
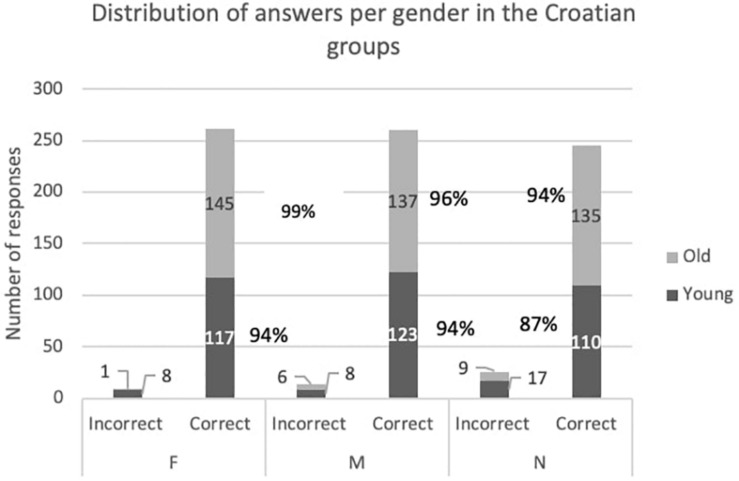
Distribution of answers per gender in the Croatian groups.

We will now look into the acquisition of F more specifically because its more prominent assignment regularity in Croatian but not in Italian might impact differently the order in which the gender values are acquired in the two languages. Figures 1, 2 show the distribution of the correct/incorrect responses divided by gender in the two languages and display the percentage of correct answers per gender and per group.

The results indicate that the Italian children are very accurate in both genders even in the young group, but nevertheless the difference between F and M is evident as the latter is at ceiling-level already in the younger group. This suggests that the earlier mastery of *la* vs. *il*, does not imply that the F gender is acquired first as a whole.

Our prediction holds for Croatian since F is the first gender to become error-free in our task. For the other two genders, the errors are still present in the older group, even if diminished. The possible reasons for this are discussed in the next section where we look at the error distribution in the two language groups. Based on this, we could argue that gender agreement in Croatian is not fully acquired in the younger group, i.e., by age 3;4 (age of oldest participant).

We have conducted ANOVAs on the distribution of answers for each gender to see how it changes with age. Table 8 displays the results of each ANOVA. It was not possible to conduct an ANOVA for M in Italian as there were only correct answers.

**TABLE 8:** ANOVAs for each gender.

**Group**	**Sum of squares**	**df**	**Mean Square**	***F***	***p***
It F	0.0510	1	0.0510	4.82	0.029
Residuals It F	3.9059	369	0.0106		
Cro F	0.220	1	0.2200	6.98	0.009
Residuals Cro F	8.481	269	0.0315		
Cro M	0.0250	1	0.0250	0.512	0.475
Residuals Cro M	13.2597	272	0.0487		
Cro N	0.344	1	0.0250	0.512	0.475
Residuals Cro N	23.162	272	0.0487		

These statistical results need to be discussed in relation to the distributions from Figures 1, 2. In Italian M is at ceiling-level already in the younger group, whereas for F a significant improvement can be noted between the age groups. When it comes to Croatian, the results have revealed a significant difference, i.e., improvement of F, likely due to the reduction of the errors from 8 to 1. The difference is not present for M as it reflects that M errors are still present in a similar proportion. When it comes to N, the group difference is significant, which means that the children have improved their agreement with N, but it still remains the gender with the most errors.

### Error Patterns

In this section, we will look a bit more closely into the errors that the children make, more precisely, which gender is used instead of the target gender. The answer is straightforward for the Italian children as no mistakes are made with M and since the language has only two genders this means that all the errors made were M agreement on a F target, which is consistent with findings from other Romance languages (Pérez-Pereira, 1991; Corrêa et al., 2011).

The error pattern in Croatian might reveal different factors at play in the two age groups. The responses are summarized in Table 9, the shaded cells marking a target-like response.

**TABLE 9:** Distribution of gender responses in Croatian children.

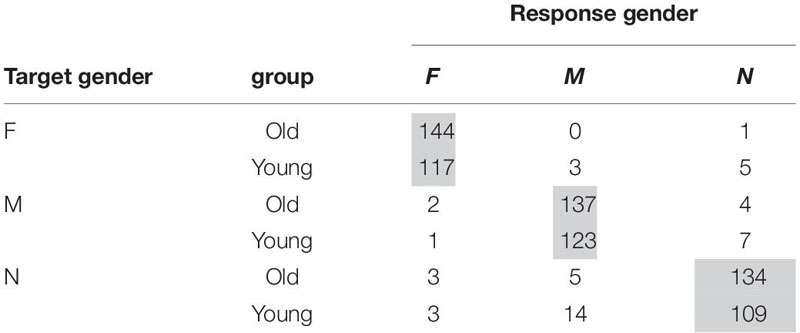

F has a similar distribution of errors among M and N. Within the errors of the other two genders it is clear how there is tendency to mistake N with M and vice versa, but F realization for both genders is also present. This tendency is likely due to the syncretism that the two genders have across the case paradigm. With respect to the age groups, M and F proceed together at an early stage, with the N lagging in accuracy. Whereas at a subsequent stage, F is acquired while M and N are at the same level. The possible reasons for this will be considered in the discussion.

## Discussion

With this task we have strived to reveal whether Italian and Croatian children differ in the time course of the acquisition of their gender system and whether individual gender values are acquired at a different pace due to the differences present in the two systems. In this section we will outline the results in relation to the literature presented throughout the paper and our predictions in order to identify the possible implications for the field of gender acquisition related to transparency.

We have predicted that the Italian children will have a higher accuracy rate due to a higher degree of transparency which is provided by (i) less ambiguous declension classes and (ii) presence of an obligatory gender-marked article which acts as a gender cue in case the noun itself does not provide it. The results confirmed this prediction as there were no significant differences between the two age groups in Italian, but there were in Croatian. This entails that the Italian children have mastered adjectival gender agreement already at age 2;6. This is in line with previous research regarding the acquisition of gender in Italian (Pizzuto and Caselli, 1992; Kupisch et al., 2002). Thus, from a theoretical perspective, the fact that the Italian gender system is morphologically transparent and has a salient gender cue on the article, makes the Italian gender system very easy to acquire. For Croatian, we cannot provide a confirmation of the mastery of adjectival gender agreement by age 3;6 due to only F being error-free. The obtained result indicates that the transparency level of e gender system is related to its acquisition and it can thus be used to make predictions related to the timing and ease of acquisition.

The results that we have obtained for Croatian are more central to the discussion, as no previous study has investigated gender acquisition in Croatian from an agreement perspective. We found that Croatian children also make few errors, which is expected as the system is transparent. However, the errors that the children made reveal that the Croatian gender system is acquired in at least two stages. These stages will be discussed in relation to our third research question below.

We have also speculated that F will be the first gender to be mastered in Croatian, due to its stronger regularity when compared to the other genders, whereas the Italian F was less ambiguous only in the article domain, and we speculated how this might not be enough to trigger an earlier mastery for the entirety of the gender value. F was indeed the first gender to be acquired in Croatian as the agreement patterns for F reach ceiling level in the older group, whereas this does not happen for the M and N. A possible reason for this finding might be (i) the syncretism of M and N in oblique cases and (ii) the considerably lower frequency of N in the input for which children require more time and exposure to grasp. In Italian F was not the first gender to be acquired, likely due to the unmarked status of M, which is a dynamic that has been observed for other Romance languages [Spanish in Pérez-Pereira (1991) and Portuguese in Corrêa et al. (2011)].

Finally, we wanted to see if the differences in the gender systems resulted in differences in the acquisition of individual genders. The Italian children are at ceiling for their adjective production at the age we tested. On the other hand, our results suggest that the Croatian children go through at least two stages which could be observed with the included age groups. The first stage consists of similar error rates with F and M, but significantly higher error rates with N. We can summarize the stage as follows: (F = M) < N. This is likely due to the lower frequency of N: 6% in CDS (Kovačević et al., 2009). The second stage consists of F being at ceiling and the error rates with M and N being similar; this also entails that the agreement accuracy for N has significantly improved since it was much more error prone than M in the younger group. This stage can be summarized like this: F < (M = N). The improvement of N agreement is probably due to a longer exposure to N nouns and their patterns. A plausible reason for M not improving as much as F is the syncretism between M and N. As the child’s exposure to and usage of the case paradigm increase, the similarity between M and N becomes more evident. If we were to test an older group, it is likely that these difficulties due to syncretism would resolve.

What the results suggest is that the degree of transparency of the gender system matters. We cannot look only at a manifestation of gender in isolation, but at the full agreement paradigm to make more accurate predictions of how a gender system might be acquired. However, the full Croatian paradigm was not investigated here as the elicitation proceeded in NOM, yet we see that the effects of syncretism in oblique cases were reflected in the agreement error rates. The Croatian gender system is acquired more gradually when compared to Italian. Nevertheless, the errors made by Croatian children are quite low in both age groups, which means that gender is grasped quite easily.

This study, among others, shows the relevance of placing transparency on a continuum. Both languages investigated here are in the higher end of the transparency scale, which makes the relative differences in transparency rather low, but these differences still lead to dissimilarities when it comes to mastery of the gender system.

We also argue that in order to evaluate the transparency of the gender system of a given language the full paradigms of the agreeing elements have to be taken into consideration. Frequent and clear cues contribute greatly to a fast mastery of the gender system (i.e., the Italian article), while syncretism leading to ambiguous gender information and low frequency in the input (Croatian N) hinder this process.

## Conclusion

This study has found differences in the time course of acquisition which can crucially be attributed to the different degrees of transparency present in Croatian and Italian. Both languages have transparent gender systems and are acquired easily. However, the gender-marked article in Italian, the syncretism of M and N in Croatian as well as the low frequency of N, make it so that the Italian system is more transparent and thus more easily acquired.

Consequently, Italian children are target-like in their realization of adjectives for both genders already in the younger group. Croatian children have overall more errors in adjectival agreement and we can recognize two stages with distinct error patterns: in the first stage they are equally accurate with F and M, and significantly less accurate with N; in the second stage F is error-free, whereas the accuracy of the M and N is roughly the same. We have attributed this to the low frequency of N, as well as the syncretism of M and N across the case paradigm. This is not a confirmation of the mastery of the full gender system in Italian, but the results nevertheless show how degree of transparency matters and how it reflects on the acquisition of gender values.

This study thus contributes to research on formal cues in gender acquisition by considering transparency as a continuum. It shows how even in two gender systems that are considered transparent; gender mastery does not proceed at an equal pace. The data presented here argues quite clearly in favor of treating transparency as more than a binary feature between transparent and opaque, and it suggests the need for a transparency scale and how to place the languages on a continuum. As we have shown here, in order to detect a language’s place on this continuum, full paradigms of the agreeing arguments should be taken into consideration. The different times of mastery of each gender value could represent a decisive starting point for future research that could include a wider age range of the participants and, more importantly, testing agreement patterns and accuracy on the full case paradigm.

Overall, even with some methodological limitations, this study found relevant results on the comparison of mastery of the gender system in two languages that differed in their degrees of transparency. This means that studying the degrees of transparency and the effects these have on the acquisition of gender is of crucial importance for the field. Future research on the acquisition of gender should consider the placement of the target language on the transparency continuum; the field should aspire to place numerous languages on continuum with well-defined parameters as this would allow us to make more accurate predictions and cross-linguistic comparisons when it comes to the acquisition of gender.

## Data Availability Statement

The dataset is available at https://marta.velnic.net/downloads/acquisition-transparent-gender-system.

## Ethics Statement

Ethical review and approval was not required for the study on human participants in accordance with the local legislation and institutional requirements. Written informed consent to participate in this study was provided by the participants’ legal guardian/next of kin.

## Author Contributions

MV collected the data, analyzed it, and wrote the full length of the manuscript.

## Conflict of Interest

The authors declare that the research was conducted in the absence of any commercial or financial relationships that could be construed as a potential conflict of interest.

## Footnotes

1 An example of a language where this overlap takes place is Spanish where the typical phonological ending for F (*-a*) is also a F morpheme that can be applied to some animate nouns (i.e., *gato*-cat/male cat vs. *gata*-female cat).

2 Similarly, also Spanish has the *-o* ending for M (singular) nouns, but this is not a morpheme in Spanish as the plural noun is formed by adding the plural morpheme *-s* to the singular form: *libro-ø/libro-s* (book/s)

3 Corpus of spoken Italian with diaphasic, diatopic and diamesic varieties.

4 Note that the Italian adjective can be placed in pre- and postnominal position (i.e., *il libro piccolo/il piccolo libro*) and some variation to meaning may apply. However, this is not the focus of the current study as the adjective is gender marked in either position.

5 Nouns in the *-i* declension class (F nouns ending in a consonant) are opaque and were not included the study.

6 The M gender additionally has an animacy sub-gender which entails a separation between M animate and inanimate nouns. These differ only in accusative singular.

7 M adjectives can be either short or long, the former ending in a consonant whereas the long ones have an additional *-i* morpheme. Both variants are clearly signalling M agreement. The distributional and semantic differences of the two variants are beyond the scope of this paper, for an overview check Aljović (2002); Trenkić (2004), Velnić (2015).

8 *L*’ is used for M and F nouns that begin with a vowel, in case of C-class nouns, the gender will be ambiguous (i.e., *l’animale*-M “animal,” *l’ape*-F “bee”).

9 Recall that N was a value only available in the Croatian groups.

## Appendix

**TABLE 1:** Distribution of the test materials across the genders in the two languages.

***English translation***	***Italian noun***	***Gender in Italian***	***Croatian Noun***	***Gender in Croatian***
*Lion*	*Leone*	*M*	*Lav*	*M*
*Frog*	*Rana*	*F*	*Žaba*	*F*
*Sun*	*Sole*	*M*	*Sunce*	*N*
*Grapes*	*Uva*	*F*	*Grožðe*	*N*
*Penguin*	*Pinguino*	*M*	*Pingvin*	*M*
*Snail*	*Lumaca*	*F*	*Puž*	*M*
*Tree*	*Albero*	*M*	*Stablo*	*N*
*Cat*	*Gatto*	*M*	*Mačka*	*F*
*Giraffe*	*Giraffa*	*F*	*Žirafa*	*F*
*Sheep*	*Pecora*	*F*	*Ovca*	*F*
*Sea*	*Mare*	*M*	*More*	*N*
*Dog*	*Cane*	*M*	*Pas*	*M*
*Butterfly*	*Farfalla*	*F*	*Leptir*	*M*
*Apple*	*Mela*	*F*	*Jabuka*	*F*
*Egg*	*Uovo*	*M*	*Jaje*	*N*
*House*	*Casa*	*F*	*Kuća*	*F*
*Mouse*	*Topo*	*M*	*Miš*	*M*
*Star*	*Stella*	*F*	*Zvijezda*	*F*
*Heart*	*Cuore*	*M*	*Srce*	*N*
*Bull*	*Toro*	*M*	*Bik*	*M*
*Ladybug*	*Cocinella*	*F*	*Bubamara*	*F*
*Moon*	*Luna*	*F*	*Mjesec*	*M*
*Eye*	*Occhio*	*M*	*Oko*	*N*
*Monkey*	*Scimmia*	*F*	*Majmun*	*M*
*Flower*	*Fiore*	*M*	*Cvijet*	*M*
*Pear*	*Pera*	*F*	*Kruška*	*F*
*Ear*	*Orecchio*	*M*	*Uho*	*N*
*Candle*	*Candela*	*F*	*Sviječa*	*F*
*Sky*	*Cielo*	*M*	*Nebo*	*N*
*Wing*	*Ala*	*F*	*Krilo*	*N*
